# Early Adoption of Services for Health-Related Social Needs in Medicare

**DOI:** 10.1001/jamahealthforum.2025.6261

**Published:** 2026-01-23

**Authors:** Jessica I. Billig, Joseph H. Joo, Jennifer R. Cardin, Michael D. Dang, Ching-Ching Claire Lin, Jim P. Stimpson, Joshua M. Liao

**Affiliations:** 1 Department of Plastic Surgery, University of Texas Southwestern Medical Center, Dallas; 2 Program on Policy Evaluation and Learning, Dallas, Texas; 3 Department of Internal Medicine, University of Texas Southwestern Medical Center, Dallas; 4 Institute of Health Policy and Management, College of Public Health, National Taiwan University, Taipei, Taiwan; 5 Department of Health Economics, Systems, and Policy, University of Texas Southwestern Medical Center, Dallas

## Abstract

This cross-sectional study assesses the provision of and reimbursement or payment denials for social determinants of health risk assessment, community health integration, and principal illness navigation services in the first year they were reimbursed by Medicare.

## Introduction

Health-related social needs (HRSNs), such as housing instability and food insecurity, are unmet nonclinical needs that can adversely affect patients’ ability to maintain health and well-being but, if addressed, can promote better outcomes.^1,2^ In 2024, Medicare began reimbursing clinicians for HRSN-related activities, including social determinants of health (SDOH) risk assessment, community health integration (CHI), and principal illness navigation (PIN).^3^ Although prior work evaluated HRSN services, this study aimed to examine their early utilization and reimbursement in Medicare.

## Methods

We used 2024 Medicare data encompassing 100% of professional services billed to and reimbursed by traditional Medicare.^4^ The University of Texas Southwestern Medical Center IRB deemed this cross-sectional study exempt from review and informed consent because it was not human participant research. We followed the STROBE reporting guideline.

Healthcare Common Procedure Coding System codes were used to capture SDOH risk assessment (code G0136), which must involve standardized, evidence-based tools that at a minimum assess for food or housing insecurity, transportation needs, and utility difficulties; CHI (codes G0019, G0022), which includes facilitating access to community-based resources, home- and community-based care coordination, and patient self-advocacy promotion; and PIN (codes G0023, G0024, G0140, G0146), which supports patients with serious, high-risk conditions expected to last at least 3 months and put patients at substantial risk of functional decline, death, or acute care utilization.

We assessed services submitted to and reimbursed or denied by Medicare. Utilization was stratified by specialty type and place of service. Specialty type included physician grouping (primary care [eg, family practice, internal medicine], medical subspecialty [eg, cardiology, oncology], and other specialty [eg, surgery]) and nonphysician grouping (advanced practice professional [eg, nurse practitioner, physician assistant] and other [eg, licensed clinical social worker]).^5^ Place of service was categorized as physician office, outpatient hospital, inpatient hospital, and other setting (eg, skilled nursing facility).

## Results

In 2024, 285 270 HRSN-related services were delivered across the US. Of those, 208 819 services (73.2%) were reimbursed through $5 007 978 in aggregate payments (Table 1). SDOH risk assessment services were most frequently delivered (82.7% [235 974]), followed by CHI (14.5% [41 362]) and PIN services (2.8% [7934]).

**Table 1.** Utilization of Services Addressing HRSN

HRSN-related service	Submitted services, No. (%)	Submitted payments, $	Denied services, No./total No. (%)	Denied payments, $	Reimbursed services, No./total No. (%)	Reimbursed payments, $
All	285 270 (100)	13 905 386	76 451/285 270 (26.8)	3 617 043	208 819/285 270 (73.2)	5 007 978
SDOH risk assessments	235 974 (82.7)	8 221 799	63 644/235 974 (27.0)	2 299 785	172 330/235 974 (73.0)	2 858 117
CHI	41 362 (14.5)	4 663 533	11 650/41 362 (28.2)	1 235 749	29 712/41 362 (71.8)	1 709 693
PIN	7934 (2.8)	1 020 054	1157/7934 (14.6)	81 508	6777/7934 (85.4)	440 168

Abbreviations: CHI, community health integration; HRSN, health-related social needs; PIN, principal illness navigation; SDOH, social determinants of health.

Most HRSN-related services were reimbursed: 73.0% (172 330) of SDOH risk assessment services, corresponding to $2 858 117 in payments; 71.8% (29 712) of CHI services, corresponding to $1 709 693 in payments; and 85.4% (6777) of PIN services, corresponding to $440 168 in payments. Of the services delivered, 26.8% (76 451) were denied and not reimbursed.

HRSN-related services were most commonly performed by primary care physicians (75.4% [215 014]), followed by advanced practice professionals (19.0% [54 372]). The minority of services were performed by medical subspecialists (3.7% [10 486]), other specialists (1.3% [3702]), and nonphysicians (0.6% [1696]) (Table 2). Most services were delivered in physician offices (87.7% [250 228]) compared with other settings (6.2% [17 741]), outpatient hospitals (3.8% [10 882]), and inpatient hospitals (2.3% [6419]).

**Table 2.** Utilization of Services Addressing Health-Related Social Needs by Specialty Type and Place of Service

	Submitted services, No. (%) (N = 285 270)	Submitted payments, $	Denied services, No./total No. (%)	Denied payments, $	Reimbursed services, No./total No. (%)	Reimbursed payments, $
**Specialty type**						
Physician grouping						
Primary care	215 014 (75.4)	10 620 990	60 833/215 014 (28.3)	2 997 021	154 181/215 014 (71.7)	3 856 979
Medical subspecialty	10 486 (3.7)	907 965	1177/10 486 (11.2)	90 962	9309/10 486 (88.8)	335 264
Other physician specialty^a^	3702 (1.3)	242 430	247/3702 (6.7)	12 360	3455/3702 (93.3)	107 170
Nonphysician grouping						
APP	54 372 (19.0)	2 106 526	13 466/54 372 (24.8)	495 715	40 906/54 372 (75.2)	703 307
Other nonphysician^b^	1696 (0.6)	27 472	728/1696 (42.9)	20 984	968/1696 (57.1)	5258
**Place of service**						
Physician office	250 228 (87.7)	12 494 157	69 685/250 228 (27.8)	3 332 008	180 543/250 228 (72.2)	4 574 480
Outpatient hospital	10 882 (3.8)	279 944	2073/10 882 (19.0)	54 349	8809/10 882 (81.0)	79 985
Inpatient hospital	6419 (2.3)	348 584	2428/6419 (37.8)	152 354	3991/6419 (62.2)	40 766
Other setting^c^	17 741 (6.2)	782 702	2265/17 741 (12.8)	78 333	15 476/17 741 (87.2)	312 747

Abbreviation: APP, advanced practice professional.

^a^ Includes emergency medicine, physical medicine and rehabilitation, psychiatry, otolaryngology, general surgery, radiation oncology, neurology, vascular surgery, interventional pain management, sports medicine, anesthesiology, urology, and surgical oncology.

^b^ Includes certified clinical nurse specialist, licensed clinical social worker, and clinic or group practice practitioners.

^c^ Includes home, assisted living facility, nursing facility, skilled nursing facility, urgent care facility, and custodial care facility.

## Discussion

In the first year Medicare reimbursed HRSN-related services, adoption was limited to the 34 million beneficiaries of traditional Medicare.^6^ Services were predominantly offered by primary care physicians in office settings, with a notable proportion of delivered services being denied by and not receiving reimbursement from Medicare. These findings highlight the critical role of primary care in supporting patients with HRSNs and the barriers to providing HRSN-related services, including misaligned infrastructure, staffing, and workflows. Denied payments also underscore that documentation requirements or other administrative burdens potentially impede adoption. Future work can identify and test interventions for reducing barriers to providing and accessing HRSN-related services.

Study limitations include the descriptive cross-sectional design and lack of practice- and patient-level data, precluding us from discussing causality of denied claims. HRSN-related services may also be coordinated outside of traditional Medicare through Medicare Advantage or state programs, especially for patients dually eligible for Medicare and Medicaid. Nonetheless, this study provides new insight into early adoption of HRSN-related services in Medicare, offering early evidence that may spur research and policy.

## Notes

Supplement.
Data Sharing Statement
