# Supplementary material for: Early Adoption of Services for Health-Related Social Needs in Medicare
Source: JAMA Health Forum. 2026 Jan 23;7(1):e256261. doi: 10.1001/jamahealthforum.2025.6261 (PMC12831150; doi:10.1001/jamahealthforum.2025.6261)
Supplement: Supplement. — Data Sharing Statement [file jamahealthforum-e256261-s001.pdf]

Data Sharing Statement

Billig. Early Adoption of Services for Health-Related Social Needs in Medicare. *JAMA Health Forum*. Published January 23, 2026. doi:10.1001/jamahealthforum.2025.6261

Data
Data available: Yes
Data types: Data (not involving human participants), Data dictionary
How to access data: Physician/Supplier Procedure Summary is a pubically available dataset. 2024 data became available on Aug 15. 2025. It is available from: https://data.cms.gov/summary-statistics-on-use-and-payments/physiciansupplier-procedure-summary
When available: beginning date: 08-15-2025

Supporting Documents
Document types: None

Additional Information
Who can access the data: Physician/Supplier Procedure Summary is a publicly available dataset. 2024 data became available on Aug 15. 2025. It is available from: https://data.cms.gov/summary-statistics-on-use-and-payments/physiciansupplier-procedure-summary
Types of analyses: For any purpose. Physician/Supplier Procedure Summary is a publicly available dataset. 2024 data became available on Aug 15. 2025. It is available from: https://data.cms.gov/summary-statistics-on-use-and-payments/physiciansupplier-procedure-summary
Mechanisms of data availability: Physician/Supplier Procedure Summary is a publicly available dataset. 2024 data became available on Aug 15. 2025. It is available from: https://data.cms.gov/summary-statistics-on-use-and-payments/physiciansupplier-procedure-summary
Any additional restrictions: N/A
